# Contralateral prophylactic mastectomy in male breast cancer: where do we stand?

**DOI:** 10.2144/fsoa-2021-0071

**Published:** 2021-07-02

**Authors:** Antonella Sciarra, Carlo Buonerba, Giuseppe Di Lorenzo, Luca Scafuri

**Affiliations:** 1 Department of Experimental Medicine, University of Campania ‘Luigi Vanvitelli’, Naples, NA, Italy; 2 Oncology Unit, Hospital ‘Andrea Tortora’, ASL Salerno, Pagani, Italy; 3 Centro di Referenza Nazionale per l'Analisi e Studio di Correlazione tra Ambiente, Animale e Uomo, Istituto Zooprofilattico Sperimentale del Mezzogiorno, Portici, Italy; 4 Associazione O.R.A., Somma Vesuviana, Naples, Italy; 5 Department of Medicine & Health Science, University of Molise, Campobasso, Italy; 6 Department of Clinical Medicine & Surgery, University of Naples ‘Federico II’, Naples, Italy

**Keywords:** contralateral prophylactic mastectomy, male breast cancer, randomized controlled trials

Similar to prostate cancer, male breast cancer (MBC) is a hormone-responsive neoplasm. Opposite to prostate cancer, MBC is extremely rare, with MBC cases accounting for <1% of all newly diagnosed malignancies and an incidence of approximately 1 in 100,000 men [1]. Importantly, temporal trends analysis performed using the Surveillance, Epidemiology, and End Results data show a 40% rise in incidence since 1975 compared with 2015 [1]. Despite the challenges posed by the rarity of this condition, multiple risk factors have been identified. Older age is a prominent risk factor, with an incidence in men ≤50 years old of 0.2 per 100,000, which climbs up to 6.3 per 100,000 in those ≥65 years old [2]. In an analysis of the prospective National Institutes of Health-AARP Diet and Health Study that involved approximately 325 thousands men including 121 with a MBC diagnosis, an approximately doubled increased risk of MBC was reported in males with a first-degree relative who had been diagnosed with breast cancer (relative risk [RR]: 1.92; 95% CI: 1.19–3.09) [3]. Of note, men who had reported a bone fracture were also at increased risk of MBC (RR: 2.20; 95% CI: 1.24–3.91), similarly to obese and inactive men [3]. A more comprehensive research conducted by analyzing a cohort of 4,501,578 men aged 18–100 years that included 642 men with primary MBC found that a number of conditions including obesity (1.98, 1.55–2.54), diabetes (RR: 1.30; 95% CI: 1.05–1.60), orchitis/epididymitis (1.84, 1.10–3.08), Klinefelter syndrome (29.64, 12.26–71.68) and gynecomastia (5.86, 3.74–9.17), were associated with MBC at univariate analysis. Among these, association of MBC with Klinefelter syndrome (16.83, 6.81–41.62), gynecomastia (5.08, 3.21–8.03), obesity (1.91, 1.50–2.44) and orchitis/epididymitis (1.80, 1.08–3.01) was also confirmed at multivariate analysis [4]. Exposure to radiation can also increase the risk of MBC, as shown by a case–control study that collected information regarding exposure to ionizing radiation in 227 cases and 300 controls and found an increased risk in men with ≥3 chest x rays and in those men who had received radiotherapy to the chest [5].

Finally, some genetic alterations may also play an important role as risk factors of MBC. In a retrospective case series of 27 MBC patients with available genetic test results, nine (33.3%) had a breast cancer gene (BRCA) 1 or 2 mutation [6]. In fact, the lifetime risk of being diagnosed with MBC is 1–5% for individuals carrying a BRCA1 alteration and 5–10% for those with a BRCA2 alteratoin, which is 10–100-times the risk estimated in the general population [7].

Mastectomy, with or without radiotherapy, hormonal therapy and chemotherapy, represents the mainstay of treatment of MBC [8]. Contralateral prophylactic mastectomy (CPM) is a highly debated option in breast cancer survivors, although it remains uncertain whether its benefits in terms of overall and cancer-free survival outweigh its cost and disadvantages [9]. Currently, presence of a BRCA mutation represents the strongest indication for CPM in both females and males. In a cross-sectional survey involving 1226 physicians among medical and radiation oncologists as well as plastic and general surgeons, 92% of participants recommended CPM in those with a mutated BRCA gene [10]. While indication for CPM in presence of a BRCA mutation is established, MBC patients who are at an ‘average’ risk of recurrence face the dilemma of undergoing CPM or not. In this regard, a significant tool has been recently made available by Li *et al.* who developed and published a nomogram capable of estimating the risk of contralateral breast cancer in male patients [11]. The researchers retrospectively analyzed the medical information of 4405 MBC patients treated with unilateral mastectomy or CPM from 1998 to 2015 retrieved from the Surveillance, Epidemiology and End Results database. The proposed nomogram was constructed to estimate the 3-, 5- and 8-year probabilities of breast cancer-specific death. The study cohort comprised 4197 patients treated with unilateral mastectomy and 208 patients treated with CPM, who were followed-up for a median of 63-months median follow-up. The authors found that the simultaneous evaluation of xi parameters including CPM, marital status, T-stage, N-stage, histology and tumor grade allowed the prediction of the pre-established outcomes with a C-index 0.75 (95% CI: 0.73–0.77) in the training cohort and 0.73 (95% CI: 0.71–0.74) in the internal validation group [11].

In this Editorial, we have reviewed useful evidence to underline the potential role of CPM in patients with MBC and the uncertainties of the associated benefits, which generates a challenging situation for both the patient and the physician. While evaluation of known risk factors, such as presence of BRCA mutation or any of the other conditions reviewed above, is surely important, the possibility of providing the exact expected increase in the chances of survival associated with CPM is of great value in the context of common clinical practice. In practical terms, we propose that in men with a BRCA mutation, CPM should always be discussed with the patient, with benefits outweighing risks in the majority of cases. In those who do not harbor a BRCA mutation, the use of the cited nomogram appears to provide valuable information to be discussed with the patient on an individual basis. For instance, in a patient with a risk of breast cancer death at 8 years of 30%, CPM can yield a considerable risk reduction in absolute terms (≅10%), which becomes trivial in men who have a risk of 5% without CPM because of the favorable characteristics of the disease. Randomized Phase III trials are required, specifically designed to randomize men who have completed the standard course of treatment for breast cancer, to CPM versus observation. Men with wild-type BRCA and men with an estimated risk of breast cancer-related death at 5 years > = 20% could be included. Although a large sample size would be needed, which may be accomplished only with an international effort, such a trial would represent a unique opportunity to establish the optimal indication for CPM.
